# Measurable residual *FLT3* tyrosine kinase domain mutations before allogeneic transplant for acute myeloid leukemia

**DOI:** 10.1038/s41409-024-02444-7

**Published:** 2024-10-18

**Authors:** Pranay S. Hegde, Georgia Andrew, Gege Gui, Niveditha Ravindra, Devdeep Mukherjee, Zoë C. Wong, Jeffery J. Auletta, Firas El Chaer, Adam Corner, Steven M. Devine, Antonio Martin Jimenez Jimenez, Marcos J. G. De Lima, Mark R. Litzow, Partow Kebriaei, Wael Saber, Stephen R. Spellman, Scott L. Zeger, Kristin M. Page, Laura W. Dillon, Christopher S. Hourigan

**Affiliations:** 1 Laboratory of Myeloid Malignancies, Hematology Branch, National Heart, Lung, and Blood Institute, National Institutes of Health, Bethesda, MD USA; 2 Fralin Biomedical Research Institute, Virginia Tech FBRI Cancer Research Center, Washington, DC USA; 3 Department of Biostatistics, Johns Hopkins Bloomberg School of Public Health, Baltimore, MD USA; 4 Center for International Blood and Marrow Transplant Research, NMDP, Minneapolis, MN USA; 5 The Ohio State University College of Medicine, Columbus, OH USA; 6 University of Virginia, Charlottesville, VA USA; 7 Bio-Rad Laboratories, Pleasanton, CA USA; 8 Sylvester Comprehensive Cancer Center, Miami, FL USA; 9 Mayo Clinic, Rochester, MN USA; 10 The University of Texas MD Anderson Cancer Center, Houston, TX USA; 11 Center for International Blood and Marrow Transplant Research, Medical College of Wisconsin, Milwaukee, WI USA

**Keywords:** Translational research, Cancer genetics, Acute myeloid leukaemia

## To the Editor:

High relapse rates for patients despite potentially curative allogeneic hematopoietic cell transplant (alloHCT) remain a critical issue in acute myeloid leukemia (AML). Next-generation sequencing (NGS) represents a promising modality for measurable residual disease (MRD) testing in patients with AML, but the appropriate targets for monitoring must be defined [1–7]. We recently reported that detection of persistent *NPM1* or *FLT3* internal tandem duplication (ITD) mutations in adults with AML in first complete remission (CR1) prior to alloHCT is associated with increased relapse and death compared with those testing negative [8]. Variants in the *FLT3* tyrosine kinase domain (TKD) are present in 7-10% of adult patients with AML at diagnosis [9], but the utility of this target for AML MRD testing prior to alloHCT is unknown

To investigate *FLT3*-TKD as an AML MRD target, we performed single-amplicon, ultra-deep, error-corrected next-generation sequencing (SA-NGS) on blood from adults in CR1 before alloHCT who were reported to have *FLT3*-TKD detected at AML diagnosis. We hypothesized that measurable residual *FLT3*-TKD variants would be associated with increased relapse and death.

Patients aged 18 or older who underwent first alloHCT for *FLT3*-TKD mutated AML in CR1 at a Center for International Blood and Marrow Transplant Research (CIBMTR) reporting site between the years of 2013 and 2019, with at least three years clinical follow-up with a suitable remission blood sample collected within 100 days prior to transplant, were eligible for this study. Nine of 351 otherwise eligible patients were excluded due to insufficient ($<$10 ng/μL or 1 μg) genomic DNA (gDNA) available. All patients gave written informed consent in accordance with the Declaration of Helsinki for participation in the CIBMTR research database (NCT01166009) and the sample repository database (NCT04920474).

SA-NGS utilizing unique molecular identifiers targeting the D835 and I836 codons of the *FLT3* gene was performed on 400 ng gDNA [10]. Libraries were sequenced (paired-end 150 bp) on a NovaSeq 6000 (Illumina) utilizing unique dual indices. Error-corrected variant calling was performed with LoFreq and the Integrative Genomics Viewer [11] and a patient was considered positive for *FLT3*-TKD MRD if the detected variant allele fraction (VAF) was above the limit of detection (LOD) threshold and greater than 0.01%. A subset of samples was orthogonally validated by droplet digital PCR (ddPCR). See also Supplemental Methods.

The primary outcomes of this analysis were cumulative incidence of relapse (CIR) and overall survival (OS) from day of transplant. Non-relapse mortality (NRM) was treated as a competing risk for CIR. Relapse-free survival (RFS) was a secondary outcome. OS and RFS were estimated with Kaplan-Meier survival analysis, and curves were compared with log-rank test. We modeled CIR using the method of Fine & Gray and compared curves with Gray’s test. Univariable analyses and multivariable analyses with stepwise selection by likelihood ratio test for OS and CIR were examined with Cox proportional hazards models for hazard ratio estimates. Statistical analysis was performed using R version 4.3.2. Serial dilution and orthogonal validation data were plotted with GraphPad Prism Version 9.5.1.

Patient, disease, and transplant characteristics for the 342 patients examined in this study are shown in Supplementary Table 1. Median age at diagnosis was 56 years (range 18–77 years) and 181 (53%) patients were female. The most common graft source was peripheral blood (*n* = 268, 78%) and the most common donor type was HLA-matched, unrelated donor (*n* = 214, 63%). Median follow-up time was 37 months (range 6–101 months) and median time to relapse was 5 months (range 1–48 months).

Using the current ELN recommended VAF threshold of 0.1% for NGS-based AML MRD detection [4], 14 patients (4.1%) tested positive for *FLT3*-TKD variants (Supplementary Fig. 1A). In univariable analyses, intermediate ELN disease risk (hazard ratio, HR 2.03, 95% CI 1.19–3.49, *p* = 0.01) and *FLT3*-TKD MRD positivity (73.8% vs. 20.5% MRD- 3-year CIR, HR 6.16, 95% CI 3.00–12.65, *p* < 0.001) were associated with increased CIR. Receipt of a cord blood graft (HR 3.35, 95% CI 1.52–7.39, *p* = 0.003) and *FLT3*-TKD MRD positivity (11.4% vs. 66.8% MRD- 3-year OS, HR 3.18, 95% CI 1.70–5.95, *p* < 0.001) were associated with decreased OS, while HLA-matched related donor transplant (HR 0.35, 95% CI 0.12–0.98, *p* = 0.046) was prognostic for increased OS.

**Fig. 1:**
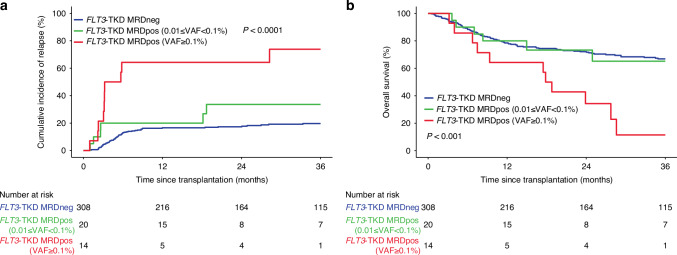
Clinical outcomes by *FLT3*-TKD MRD status. Rates of (**a**) relapse and (**b**) overall survival for patients based on the presence (MRDpos) or absence (MRDneg) of residual *FLT3* tyrosine kinase domain (TKD) variants in pre-transplant blood. *FLT3*-TKD MRDpos patients were divided by the level of residual disease based on variant allele fraction (VAF) above or below 0.1%. Survival curves plotted using the Kaplan-Meier estimator and compared with the log-rank test and cumulative incidence of relapse plotted using the cumulative incidence function and compared with Gray’s test.

Using the highly sensitive SA-NGS method, we were able to explore if a deeper VAF threshold of 0.01% was prognostic for *FLT3*-TKD as an MRD target. An additional 20 patients were reclassified as positive (*n* = 34 with VAF$\ge$0.01%, 9.9%) (Supplementary Fig. 1B). However, for patients with VAF between 0.01% and 0.1%, there were no statistically significant differences in CIR (33.6% vs. 19.7% MRD- 3-year CIR, HR 2.00, 95% CI 0.90–4.42, *p* = 0.087) and OS (65.2% vs. 66.9% MRD- 3-year CIR, HR 1.48, 95% CI 0.75–2.92, *p* = 0.264) compared to VAF$<$0.01% (Fig. 1). Site-reported pre-transplant CR1 multiparametric flow cytometry results were available for 335 (98%) patients and were not associated with either CIR (HR 1.3, 95% CI 0.56–3.02, *p* = 0.54) or OS (HR 0.78, 95% CI 0.34–1.77, *p* = 0.551, Supplementary Fig. 2).

Orthogonal validation with ddPCR was performed for patients with D835Y, D835H, and D835V VAFs$\ge$0.01% (*n* = 28). Six (21%) SA-NGS positive calls at this threshold could not be validated, and none of these patients experienced relapse. In multivariable analyses, only detected *FLT3-*TKD VAF$\ge$0.1% was associated with both increased CIR (HR 6.16, 95% CI 3.00–12.65, *p* < 0.001) and death (HR 3.17, 95% CI 1.69–5.94, *p* < 0.001).

In this study, we report the prognostic significance of pre-transplant detectable *FLT3*-TKD variants in blood from adult AML patients in CR1 prior to alloHCT for relapse and survival. Though SA-NGS allowed us to examine the VAF cutoff of 0.01% used in the Pre-MEASURE study for *FLT3-*ITD and *NPM1* [7], a significant prognostic association was only seen for VAF$\ge$0.1%. This highlights that optimal VAF cutoffs for AML MRD should be determined specifically based on individual targets and test characteristics, in addition to tissue type, timepoint, and treatment type. Orthogonal validation for ddPCR was not successful for 21% of patients tested, and their lack of subsequent relapse may suggest they were analytical false positive results. Alternative NGS technology better suited for rare single nucleotide variant detection may have utility for this target [12].

This study has several limitations. Our use of a single-amplicon NGS assay did not allow determination of the persistence of other potential AML MRD targets. The rarity of *FLT3*-TKD persistence in CR1 (seen in 4–10% of our samples depending on methodology and VAF cutoff), provided insufficient power for subgroup analyses. Additionally, diagnostic and relapse samples, information on pre-transplant therapy choice, and pre- and post-transplant *FLT3* inhibitor use were not available for analysis in this retrospective registry biobank study. It has recently been shown that post-transplant maintenance with a *FLT3* inhibitor can mitigate the prognostic significance of pre-transplant MRD in *FLT3*-ITD patients [13], but the significance has yet to be determined in *FLT3*-TKD mutated patients.

This work provides evidence that persistence of detectable *FLT3*-TKD variants in pre-alloHCT CR1 blood is rare but associated with increased relapse and decreased overall survival using a VAF cutoff of 0.1%. Optimal NGS-MRD testing for patients with AML will likely require a multi-target approach. Testing for persistence of *FLT3*-TKD MRD is shown here to represent an important component of this strategy.

## Supplementary information

Supplementary Material

## Data Availability

Sequencing data are available in the NCBI Sequence Read Archive (SRA) database (Accession: PRJNA979814). Clinical data will be published by CIBMTR as a resource.

## References

[1] Loo S, Dillon R, Ivey A, Anstee NS, Othman J, Tiong IS, et al. Pretransplant FLT3-ITD MRD assessed by high-sensitivity PCR-NGS determines posttransplant clinical outcome. Blood. 2022;140:2407–11. 10.1182/blood.2022016567 35960851 10.1182/blood.2022016567 PMC10653044

[2] Lee JM, Park S, Hwang I, Kang D, Cho BS, Kim HJ. et al. FLT3-ITD measurable residual disease monitoring in acute myeloid leukemia using next-generation sequencing. Cancers (Basel). 2022;14. 10.3390/cancers14246121 10.3390/cancers14246121 PMC9776673 36551616

[3] Hourigan CS, Dillon LW, Gui G, Logan BR, Fei M, Ghannam J, et al. Impact of conditioning intensity of allogeneic transplantation for acute myeloid leukemia with genomic evidence of residual disease. J Clin Oncol. 2020;38:1273–83. 10.1200/JCO.19.03011 31860405 10.1200/JCO.19.03011 PMC7164487

[4] Heuser M, Freeman SD, Ossenkoppele GJ, Buccisano F, Hourigan CS, Ngai LL, et al. 2021 Update on MRD in acute myeloid leukemia: a consensus document from the European LeukemiaNet MRD Working Party. Blood. 2021;138:2753–67. 10.1182/blood.2021013626 34724563 10.1182/blood.2021013626 PMC8718623

[5] Grob T, Sanders MA, Vonk CM, Kavelaars FG, Rijken M, Hanekamp DW, et al. Prognostic value of FLT3-internal tandem duplication residual disease in acute myeloid leukemia. J Clin Oncol. 2023;41:756–65. 10.1200/JCO.22.00715. 36315929 10.1200/JCO.22.00715 PMC9901965

[6] Ghannam J, Dillon LW, Hourigan CS. Next-generation sequencing for measurable residual disease detection in acute myeloid leukaemia. Br J Haematol. 2020;188:77–85. 10.1111/bjh.16362. 31804716 10.1111/bjh.16362

[7] Dillon LW, Gui G, Page KM, Ravindra N, Wong ZC, Andrew G, et al. DNA sequencing to detect residual disease in adults with acute myeloid leukemia prior to hematopoietic cell transplant. JAMA. 2023;329:745–55. 10.1001/jama.2023.1363 36881031 10.1001/jama.2023.1363 PMC9993183

[8] Dillon LW, Gui G, Ravindra N, Andrew G, Mukherjee D, Wong ZC, et al. Measurable residual FLT3 internal tandem duplication before allogeneic transplant for acute myeloid leukemia. JAMA Oncol. 2024. 10.1001/jamaoncol.2024.0985. 38696205 10.1001/jamaoncol.2024.0985 PMC11066770

[9] Tyner JW, Tognon CE, Bottomly D, Wilmot B, Kurtz SE, Savage SL, et al. Functional genomic landscape of acute myeloid leukaemia. Nature. 2018;562:526–31. 10.1038/s41586-018-0623-z. 30333627 10.1038/s41586-018-0623-z PMC6280667

[10] Thol F, Gabdoulline R, Liebich A, Klement P, Schiller J, Kandziora C, et al. Measurable residual disease monitoring by NGS before allogeneic hematopoietic cell transplantation in AML. Blood. 2018;132:1703–13. 10.1182/blood-2018-02-829911. 30190321 10.1182/blood-2018-02-829911 PMC7116653

[11] Robinson JT, Thorvaldsdottir H, Winckler W, Guttman M, Lander ES, Getz G, et al. Integrative genomics viewer. Nat Biotechnol. 2011;29:24–6. 10.1038/nbt.1754 21221095 10.1038/nbt.1754 PMC3346182

[12] Schmitt MW, Kennedy SR, Salk JJ, Fox EJ, Hiatt JB, Loeb LA. Detection of ultra-rare mutations by next-generation sequencing. Proc Natl Acad Sci USA. 2012;109:14508–13. 10.1073/pnas.1208715109. 22853953 10.1073/pnas.1208715109 PMC3437896

[13] Levis MJ, Hamadani M, Logan B, Jones RJ, Singh AK, Litzow M, et al. Gilteritinib as post-transplant maintenance for AML with internal tandem duplication mutation of FLT3. J Clin Oncol. 2024;42:1766–75. 10.1200/JCO.23.02474. 38471061 10.1200/JCO.23.02474 PMC11095884

