# Supplementary material for: Measurable residual FLT3 tyrosine kinase domain mutations before allogeneic transplant for acute myeloid leukemia
Source: Bone Marrow Transplant. 2024 Oct 18;60(2):175–7. doi: 10.1038/s41409-024-02444-7 (PMC11810768; doi:10.1038/s41409-024-02444-7)
Supplement: Supplementary file 1 — Supplementary Material [file 41409_2024_2444_MOESM1_ESM.pdf]

Measurable Residual *FLT3* Tyrosine Kinase Domain Mutations before Allogeneic Transplant for Acute Myeloid Leukemia

Pranay S. Hegde[1], Georgia Andrew[1], Gege Gui[2,3], Niveditha Ravindra[1], Devdeep Mukherjee[1], Zoë C. Wong[1], Jeffery J. Auletta[4,5], Firas El Chaer[6], Adam Corner[7], Steven M. Devine[4], Antonio Martin Jimenez Jimenez[8], Marcos J. G. De Lima[5], Mark R. Litzow[9], Partow Kebriaei[10], Wael Saber[11], Stephen R. Spellman[4], Scott L. Zeger[3], Kristin M. Page[11], Laura W. Dillon[1*], Christopher S. Hourigan[2*]

[1]Laboratory of Myeloid Malignancies, Hematology Branch, National Heart, Lung, and Blood Institute, National Institutes of Health, Bethesda, MD
[2]Fralin Biomedical Research Institute, Virginia Tech FBRI Cancer Research Center, Washington, DC
[3]Department of Biostatistics, Johns Hopkins Bloomberg School of Public Health, Baltimore, MD
[4]Center for International Blood and Marrow Transplant Research, NMDP, Minneapolis, MN
[5]The Ohio State University College of Medicine, Columbus, OH
[6]University of Virginia, Charlottesville, VA
[7]Bio-Rad Laboratories, Pleasanton, CA
[8]Sylvester Comprehensive Cancer Center, Miami, FL
[9]Mayo Clinic, Rochester, MN
[10]The University of Texas MD Anderson Cancer Center, Houston, TX
[11]Center for International Blood and Marrow Transplant Research, Medical College of Wisconsin, Milwaukee, WI
* These authors contributed equally.

Table of Contents

Supplementary Methods	Page 2
Supplementary Figure 1: *FLT3*-TKD variants identified in AML patients during remission	Page 9
Supplementary Figure 2: Site reported flow cytometry results and clinical outcomes	Page 10
Supplementary Table 1: Patient characteristics	Page 11
Supplementary Table 2: *FLT3*-TKD variants identified by SA-NGS and ddPCR in AML patients during remission	Page 13
Supplementary Table 3: Primers for SA-NGS of *FLT3*-TKD	Page 14
References	Page 15

Supplementary Methods:

Extraction of Genomic DNA (gDNA)

Pre-transplant remission peripheral blood (PB) samples were available for 351 patients known to be positive for *FLT3* tyrosine kinase domain (TKD) variants at diagnosis. High-quality gDNA was extracted using the Chemagic® DNA Blood 2k kit H24 (cat# CMG-1097, PerkinElmer Health Sciences Inc., Shelton, CT) on the chemagic360 instrument (PerkinElmer Health Sciences Inc., Shelton, CT). DNA concentration was determined fluorometrically using the Quant-iT PicoGreen® dsDNA Assay kit (cat# P11496, ThermoFisher Scientific, Waltham, MA) on the Synergy LX microplate reader (BioTek Instruments Inc., Winooski, VT). A median of 16.8 μg (range 0 – 76.5 μg) was obtained, with 342 (97.4%) patients having sufficient DNA for next generations sequencing (NGS) library preparation. Samples too dilute for evaluation (< 10 ng/μL) were concentrated using SPRISelect beads (cat #B23318, Beckman Coulter Inc., Indianapolis, IN).

Single-Amplicon Next-Generation Sequencing

Single-amplicon targeted, error-corrected, next-generation sequencing (SA-NGS) was performed on gDNA from pre-transplant PB of 342 *FLT3*-TKD mutated AML patients utilizing a protocol modified from Thol *et al*[1]. For each sample, 200ng of gDNA was amplified in duplicate (400ng total per patient) with primers (TKD-F and TKD-R, **Supplementary Table 3**) containing 16-nt unique molecular identifiers (UMIs) targeting the D835 and I836 codons of the *FLT3*-TKD locus by incubating with the high-fidelity Q5 DNA polymerase (New England Biolabs Inc., Ipswich, MA) in a thermal cycler as follows: 98°C for 30 sec; 5 cycles of 98°C for 10 sec, 60°C for 50 sec, 72°C for 12 sec; 72°C for 2 min. DNA was purified using a two-step protocol with SPRISelect beads (Beckman Coulter) at a 0.9:1 bead:PCR product ratio, followed by a 0.21:1 ratio and eluted in nuclease-free water. The eluent was used for the indexing PCR reaction (SA-NGS-P5 and SA-

NGS-P7, **Supplementary Table 3**) by incubating with Q5 DNA polymerase in a thermal cycler as follows: 98°C for 30 sec; 25 cycles of 98°C for 10 sec, 67°C for 50 sec, 72°C for 12 sec; 72°C for 2 min. Products were purified using a SPRISelect bead purification at 0.55:1 and 0.9:1 bead:PCR product ratios and eluted in nuclease-free water.

Final SA-NGS library concentrations were determined fluorometrically using the Qubit dsDNA high-sensitivity assay kit (cat #Q32851, Thermofisher Scientific, Waltham, MA) on the Qubit® 3.0 Fluorometer (cat #Q33216 ThermoFisher Scientific, Waltham, MA). Equimolar concentrations of up to 96 samples were pooled and average library size was determined using the HS D1000 screentape on the TapeStation 4200 instrument (Agilent Technologies, Inc). SA-NGS libraries were subjected to paired-end 150-bp sequencing on the NovaSeq 6000 instrument (Illumina).

Raw FASTQ files are available in the NCBI Sequence Read Archive (SRA) database (Accession: PRJNA979814).

Patient *FLT3*-TKD libraries were prepared in batches including gDNA healthy donor controls known to be negative for *FLT3*-TKD mutations (negative control). A total of 36 sample replicates from three healthy donors were analyzed across the library preparation batches. False-positive negative controls in subsequent analyses triggered repeat library preparation for the entire batch. Two batches of eight samples were repeated following these criteria.

Bioinformatic sequencing data processing and analysis

Processing of raw fastq sequencing files was performed as previously detailed by Thol *et al*[1], with slight modifications. Briefly, sequencing reads were aligned to the target sequence derived from human genome hg19 using bwa (Burrow-Wheeler Aligner, algorithm option "aln+samse", "single-end"). This was performed after using the Paired End ReAd MergeR (PEAR)[2] to assemble the

forward (R1) and reverse (R2) reads, and a third bam file (R1/R2) was generated by aligning matching bases on forward and reverse reads and aligning to the reference sequence.

For single nucleotide variants (SNVs), an error-corrected, read family-derived bam file (RF) was generated using a series of duplex sequencing data analysis scripts previously referenced[1]. The sequencing reads were trimmed to their UMI, with the forward and reverse UMIs being used to define sequence headers. The two sets of reads were then aligned to the reference sequence, and the ConsensusMaker.py script was applied to generate the RF bam file. Each RF file consists of single-strand consensus sequences that represent read families identified by a unique UMI. Read families with fewer than 3 sequences were removed from further analysis for insufficient depth. A nucleotide congruence of >70% between reads was required to assign a base to the single-strand consensus sequence, with positions failing to meet this criterion receiving an "N" not used in variant calling.

The RF bam file was analyzed with the Integrative Genomics Viewer (University of California San Diego, La Jolla, CA) and base counts were produced at each target position and used to determine allele fractions. The largest non-reference, or variant, allele fraction (LVAF) at each position was recorded. The average and standard deviation (SD) of LVAFs was determined over all non-primer region positions, with removal of LVAFs greater than 2.5 standard deviations above the average. The average and SD were re-calculated from this subset of LVAFs and represented the mean background error (MBE) determined from 40 positions around the target mutation, with the target being the center or shifted depending on its distance to the primer region. Those within 5bp of the primer were removed from the background calculation. The limit of detection (LOD) for each relevant nucleotide position was calculated in each sample as the MBE plus 3 standard deviations. Variants were matched with the appropriate amino acid substitution using a data dictionary that included expected nucleotide changes and their predicted variants.

Insertion or deletion (indel) mutations were extracted from the above analysis, with indels being pulled with the Samtools mpileup utility[3]. Detected variants were manually curated for pathogenicity, with only in-frame deletions considered.

Establishment of measurable residual disease (MRD) criteria

To detect SNVs as low as a rate of 1 in 10,000 (0.01% VAF), we required samples to have at least 10,000 read families to be evaluable. 100% of samples sequenced in this study met this criterion. Samples were considered MRD positive by SNV criteria if:

1. Target position LVAF >LOD
2. Target position LVAF ≥0.01%
3. No other peak within a maximum of 20bp of the target peak > target position LVAF minus 3 standard deviations of background error. If primer sequences were within 20 bp of the target position, this criterion was applied up to the nucleotide prior to the primer sequence.
4. If multiple LVAFs above the LOD were found in close proximity within a sample, LOD was determined for each variant after removing the other from the LOD calculation. This ensured that the LOD was not falsely elevated by the presence of two variants within a sample. Additionally, MRD positive rate was not affected, but the LOD was more accurate for samples with multiple neighboring variants.

For indels, samples were considered MRD positive if:

1. The variant was an in-frame insertion or deletion of the 835 or 836 codons.
2. Indel VAF ≥0.01%, where the variant count is determined from the R1/R2 aligned read file.

Patients positive for MRD by both SNV and indel criteria were treated as MRD positive. MRD status for each sample was documented prior to accession and analysis of clinical outcomes.

Droplet Digital PCR Validation of SA-NGS Data:

Patients with missense mutations for which assays were available (D835Y, D835V, D835H) were selected for orthogonal validation with droplet digital PCR (ddPCR). This encompassed a subset of 28 of 34 SA-NGS identified variants (82.4%) with VAFs ranging from 0.01% to 62.2%. DNA was evaluated using allele-specific fluorescent TaqMan probe assays labeled with FAM or HEX fluorophores in accordance with manufacturer specifications (ddPCR mutation detection assay, cat #10049550, Bio-Rad Laboratories Inc., Hercules, CA) on the QX200 instrument. DNA was incubated with the HindIII restriction enzyme to improve PCR yield. Samples with anticipated VAF $< 0.1\%$ were run in triplicate with 110 ng gDNA per well and merged for analysis. Samples with anticipated VAF $\geq 0.1\%$ were run in a single well each with 110 ng DNA. Positive and negative control samples were used to apply thresholds and limit of detection cut-offs using a 95% confidence interval based on a Poisson distribution. As MRD status was locked using SA-NGS prior to validation with ddPCR, analyses using orthogonal information from this assay are demarcated as such.

Conditioning Intensity:

Patients were divided into 3 categories of conditioning treatment regimens prior to hematopoietic cell transplantation using the following criteria:

1. Myeloablative conditioning (MAC)
    a. Total body irradiation (TBI) >500 cGy, non-fractionated or TBI >800 cGy, fractionated
    b. Busulfan and Cyclophosphamide

    c. Busulfan and Melphalan
    d. Busulfan and Thiotepa
    e. Cyclophosphamide and Thiotepa
    f. Busulfan (≥9 mg/kg, oral or ≥7.2 mg/kg, IV) and Fludarabine
2. Reduced intensity conditioning (RIC)
    a. TBI ≤500 cGy but >200cGy, non-fractionated or TBI ≤800 cGy but >200cGy, fractionated
    b. Melphalan and Fludarabine
    c. Busulfan (<9 mg/kg, oral or <7.2 mg/kg, IV) and Fludarabine
    d. Cyclophosphamide and BNCU and Etoposide (VP-16) (CBV)
    e. BCNU + Etoposide (VP-16) + Cytarabine (Ara-C) + Melphalan (BEAM)
3. Non-myeloablative conditioning (NMA)
    a. TBI = 200cGy
    b. Fludarabine with or without Cyclophosphamide
    c. Total lymphoid irradiation (TLI) alone

There were 25% (n=50) patients with Bu/Cy, 55% (n=109) with Bu/Flu, and 20% (n=40) with other regimens for patients having MAC. There were 17% (n=24) patients with Cy/Flu, 31% (n=45) with Bu/Flu, 42% (n=60) with Mel/Flu, and 10% (n=14) with other regimens for patients having RIC/NMA.

<u>Statistical Analysis:</u>

Clinical data for the 342 patients was obtaining from the Center for International Blood and Marrow Transplant Research (CIBMTR) database. For clinical outcomes, day 0 was the day of hematopoietic cell transplantation. The primary outcome was overall survival (OS), with secondary endpoints including cumulative incidence of relapse (CIR), and relapse-free survival

(RFS). Non-relapse mortality (NRM) was included as a competing risk. Relapse time was defined as the time (in months) between alloHCT and morphologic relapse, while relapse-free survival was the time from alloHCT to relapse or death of any cause. OS and RFS endpoints were determined using the Kaplan-Meier estimator, and curves were compared with the log-rank test. We examined CIR with NRM as a competing risk using Fine & Gray regression and compared curves with Gray's test.

Univariate analysis was conducted on baseline characteristics using unadjusted Cox models (OS) and competing risk models (CIR). Multivariable analysis included Cox/competing risk regression models for OS and relapse, with variables selected for inclusion in the models from the available clinical characteristics using stepwise selection. Survival and regression analyses were carried out in R version 4.3.0 (2023).

Supplementary Figure 1. *FLT3*-TKD variants identified in AML patients during remission. The frequency (left) and variant allele fraction (VAF, right) of *FLT3*-TKD variants detected in remission by SA-NGS displayed as amino acid substitutions expected from the relevant nucleotide changes for (A) VAF ≥0.1% and (B) VAF ≥0.01%. Δ = deletion. Horizontal bars represent the median VAF for each variant.

A

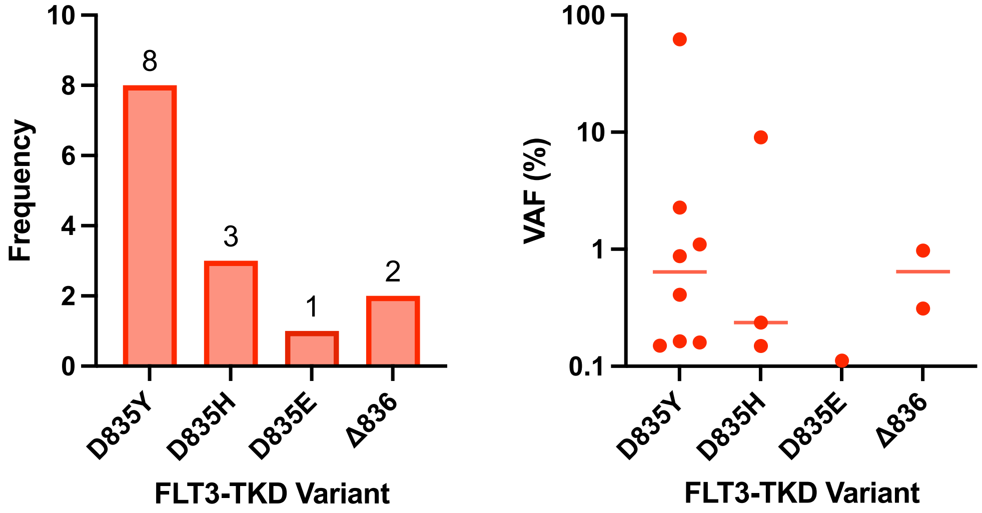

B

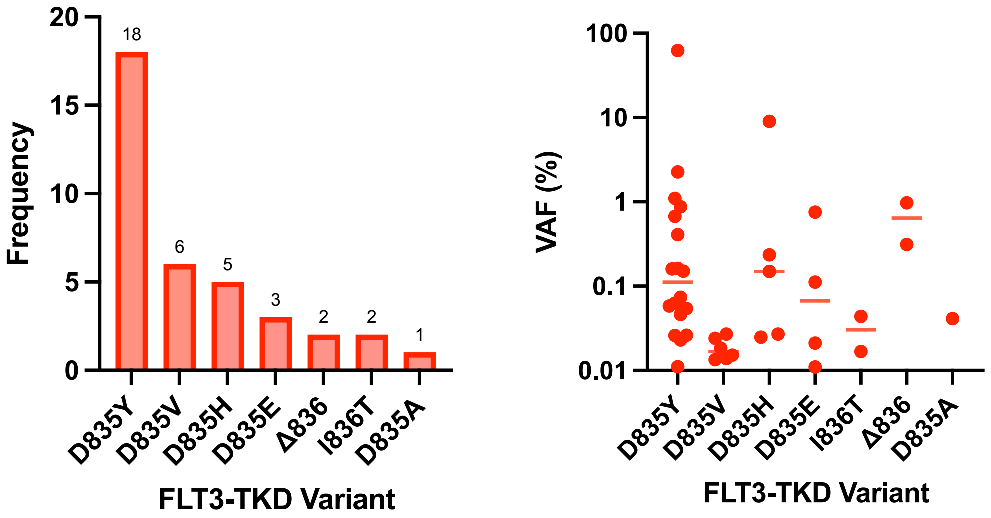

Supplementary Figure 2: Association of site flow cytometry and clinical outcomes for patients with *FLT3*-TKD AML. Rates of (A) relapse, (B) overall survival, (C) relapse-free survival, and (D) non-relapse mortality (NRM) shown at 36 months for patients with data available for residual disease by flow cytometry as reported by the treatment site. Survival curves plotted using the Kaplan-Meier estimator and compared with the log-rank test. Cumulative incidence of relapse and NRM plotted using the cumulative incidence function and compared with Gray's test.

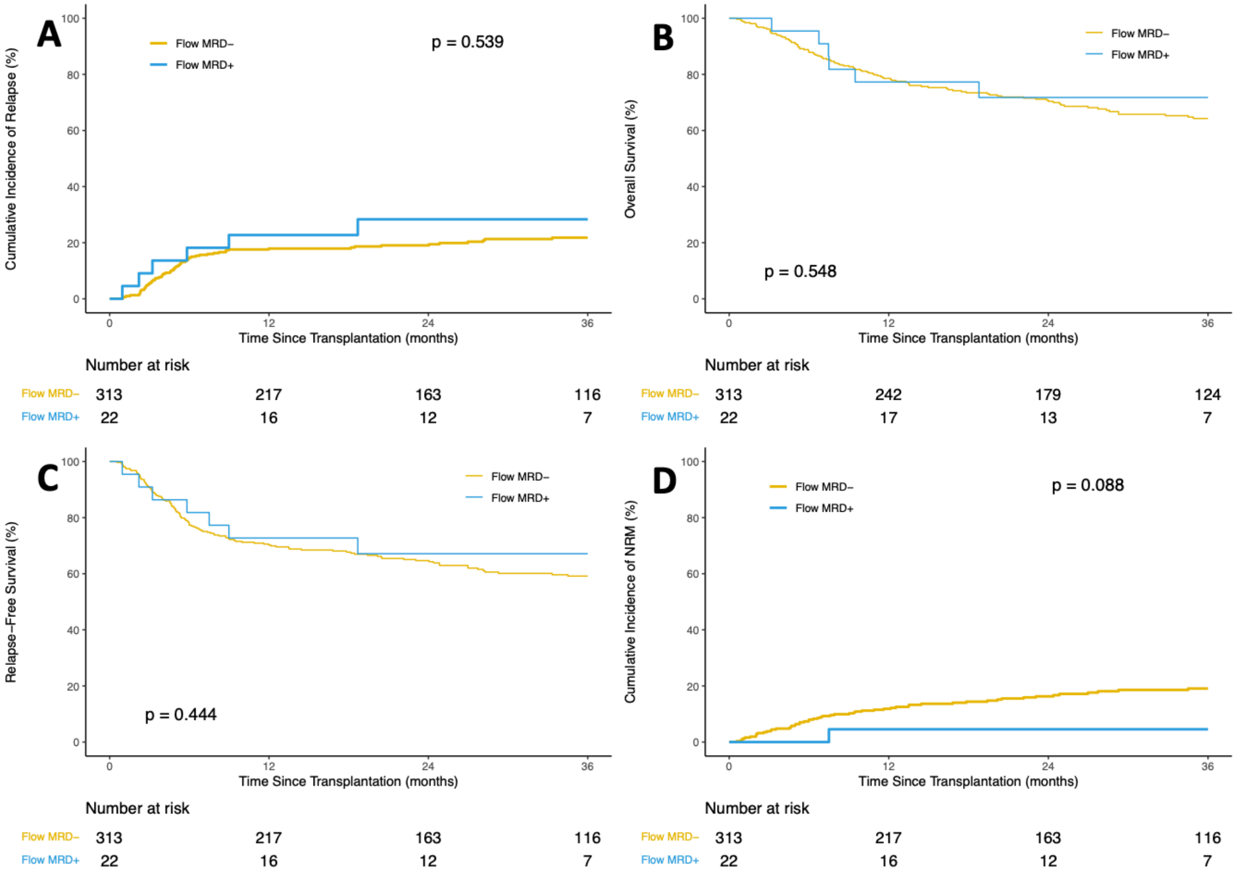

Supplementary Table 1: Patient characteristics. Continuous variables reported as mean (standard deviation) and median [minimum, maximum].

	***FLT3*-TKD MRD negative (N=328)**	***FLT3*-TKD MRD positive (N=14)**	**Overall (N=342)**
**Age (years)**			
Mean (SD)	53.1 (13.3)	57.3 (13.2)	53.3 (13.3)
Median [Min, Max]	56.2 [18.8, 77.6]	63.4 [29.3, 72.5]	56.2 [18.8, 77.6]
**Sex**			
Female	174 (53.0%)	7 (50.0%)	181 (52.9%)
**Race**			
Other/Unknown	41 (12.5%)	2 (14.3%)	43 (12.6%)
White	287 (87.5%)	12 (85.7%)	299 (87.4%)
**Survival Status**			
Alive w/o Disease	197 (60.1%)	2 (14.3%)	199 (58.2%)
Relapse	69 (21.0%)	10 (71.4%)	79 (23.1%)
Non-Relapse Mortality	62 (18.9%)	2 (14.3%)	64 (18.7%)
**AML Type**			
De novo	304 (92.7%)	9 (64.3%)	313 (91.5%)
Therapy-linked	14 (4.3%)	4 (28.6%)	18 (5.3%)
Transformed MDS/MPN	10 (3.0%)	1 (7.1%)	11 (3.2%)
**ELN Disease Risk**			
Adverse	64 (19.5%)	5 (35.7%)	69 (20.2%)
Favorable	125 (38.1%)	1 (7.1%)	126 (36.8%)
Intermediate	139 (42.4%)	8 (57.1%)	147 (43.0%)
**Graft Type**			
Peripheral Blood	256 (78.0%)	12 (85.7%)	268 (78.4%)
Cord Blood	27 (8.2%)	1 (7.1%)	28 (8.2%)
Bone Marrow	45 (13.7%)	1 (7.1%)	46 (13.5%)
**Karnofsky Performance Status**			
≥90	198 (60.4%)	7 (50.0%)	205 (59.9%)
**HCT-Comorbidity Index**			
0	62 (18.9%)	0 (0%)	62 (18.1%)
1-2	94 (28.7%)	5 (35.7%)	99 (28.9%)
≥3	172 (52.4%)	9 (64.3%)	181 (52.9%)
**Donor Group**			
Cord Blood	19 (5.8%)	1 (7.1%)	20 (5.8%)
Haploidentical Related	24 (7.3%)	1 (7.1%)	25 (7.3%)
HLA-Matched Related	49 (14.9%)	0 (0%)	49 (14.3%)
HLA-Matched Unrelated	204 (62.2%)	10 (71.4%)	214 (62.6%)
Mismatched	24 (7.3%)	2 (14.3%)	26 (7.6%)
Multiple Donor	8 (2.4%)	0 (0%)	8 (2.3%)
**Conditioning Regimen**			
Myeloablative	190 (57.9%)	9 (64.3%)	199 (58.2%)
Reduced intensity/non-myeloablative	138 (40.4%)	5 (1.5%)	143 (41.8%)
**Anti-Thymocyte Globulin Usage**			
Yes	98 (29.9%)	5 (35.7%)	103 (30.1%)
***FLT3*-ITD mutated**			

Yes	108 (32.9%)	4 (28.6%)	112 (32.7%)
***NPM1* mutated**			
Yes	168 (51.2%)	2 (14.3%)	170 (49.7%)

TKD, tyrosine kinase domain; MRD, measurable residual disease; MDS, myelodysplastic syndrome; MPN, myeloproliferative neoplasm; AML, acute myeloid leukemia; ELN, European LeukemiaNet; HLA, human leukocyte antigen; ITD, internal tandem duplication

Supplementary Table 2: *FLT3*-TKD variants identified by SA-NGS and ddPCR in AML patients during remission

Patient ID	*FLT3* Mutation	SA-NGS VAF (%)	ddPCR VAF (%)
CH015525	D835E	0.021	Not tested
CH015166	D835Y	2.265	2.5289
CH015166	D835E	0.753	Not tested
CH015166	D835E	0.011	Not tested
CH015212	D835H	9.043	8.266
CH015212	D835V	0.024	Not tested
CH015339	D835V	0.014	0.0341
CH015498	D835V	0.018	0.016
CH015679	D835H	0.150	0.1712
CH015820	D835Y	0.023	0.0247
CH015923	Δ836	0.974	Not tested
CH015923	D835Y	0.675	0.6363
CH015972	D835Y	0.410	0.4698
CH012210	D835Y	62.153	60.5
CH012460	D835Y	0.026	0.0328
CH012222	D835Y	0.063	Not detected
CH016999	D835Y	0.054	0.0773
CH016810	D835Y	0.150	Not detected
CH016436	D835Y	0.160	Not detected
CH016477	D835H	0.027	0.0385
CH016626	D835Y	0.058	Not detected
CH016528	D835Y	0.046	Not detected
CH016573	D835V	0.027	0.0097
CH016869	D835E	0.112	Not tested
CH017055	D835Y	1.097	1.2482
CH012023	D835Y	0.011	Not detected
CH012395	D835A	0.041	Not tested
CH017178	D835Y	0.074	0.1048
CH017317	D835Y	0.026	0.0145
CH017512	D835V	0.013	0.0246
CH017540	D835V	0.015	0.0279
CH017658	D835H	0.236	0.2703
CH012449	D835Y	0.871	0.848
CH012363	I836T	0.044	Not tested
CH012036	I836T	0.017	Not tested
CH016474	D835Y	0.163	0.116
CH017420	D835H	0.025	0.0213
CH012254	Δ836	0.312	Not tested

Single amplicon next generation sequencing, SA-NGS
Digital droplet PCR, ddPCR
Variant allele fraction, VAF

Supplementary Table 3. Primers for SA-NGS of *FLT3*-TKD.

Primer Name	Sequence (5'-3')
TKD-F	GGTAAACACAAGGGCACTGGNNNNNNNNNNNNNNNNCAGGAACGTGCTTGTCACC
TKD-R	CGGACTACAGCTCCCATCATNNNNNNNNNNNNNNNNCATTGCCCCTGACAACATAG
SA-NGS-P5	AATGATACGGCGACCACCGAGATCTACACNNNNNNNNACACTCTTTCCCTACACGACGCTCTTCCGATCTNGGTAAACACAAGGGCACTGG
SA-NGS-P7	CAAGCAGAAGACGGCATACGAGATNNNNNNNNGTGACTGGAGTTCAGACGTGTGCTCTTCCGATCTNCGGACTACAGCTCCCATCAT

Blue, 16-nt UMI; Red, Illumina P5 index; Green, Spacer (range 1-4nt); Orange, Illumina P7 index

References

1. Thol F, Gabdoulline R, Liebich A, Klement P, Schiller J, Kandziora C *et al.* Measurable residual disease monitoring by NGS before allogeneic hematopoietic cell transplantation in AML. *Blood* 2018; **132**(16)**:** 1703-1713. e-pub ahead of print 20180906; doi: 10.1182/blood-2018-02-829911

2. Zhang J, Kobert K, Flouri T, Stamatakis A. PEAR: a fast and accurate Illumina Paired-End reAd mergeR. *Bioinformatics* 2014; **30**(5)**:** 614-620. e-pub ahead of print 20131018; doi: 10.1093/bioinformatics/btt593

3. Danecek P, Bonfield JK, Liddle J, Marshall J, Ohan V, Pollard MO *et al.* Twelve years of SAMtools and BCFtools. *Gigascience* 2021; **10**(2). doi: 10.1093/gigascience/giab008
